# “Take me seriously and do something!” - a qualitative study exploring patients’ perceptions and expectations of an upcoming orthopaedic consultation

**DOI:** 10.1186/s12891-017-1719-6

**Published:** 2017-08-24

**Authors:** Karin S Samsson, Susanne Bernhardsson, Maria EH Larsson

**Affiliations:** 1 University of Gothenburg, Institute of Neuroscience and Physiology at Sahlgrenska Academy , Department of Health and Rehabilitation, Box 430, 405 30 Gothenburg, Sweden; 2 Närhälsan Tjörn Rehabilitation Clinic, Primary Health Care, Region Västra Götaland, Syster Ebbas väg 1, 471 94 Kållekärr, Sweden; 3 Närhalsan Research and Development Primary Health Care, Region Västra Götaland, Kungsgatan 12, 6th floor, 411 18 Gothenburg, Sweden

**Keywords:** Perceptions, Expectations, Musculoskeletal disorders, Orthopaedic, Content analysis

## Abstract

**Background:**

Patients’ perceptions of care is an important factor in evaluation of health care, in quality assessment, and in improvement efforts. Expectations of assessments or procedures such as surgery have been found to be related to perceptions of outcome as well as satisfaction, and are therefore of interest to both clinicians and researchers. Increased understanding of these patient views is important so that orthopaedic assessments, regardless of who performs them, can be further developed and patient-centred to better meet patients’ needs. The purpose of this study was therefore to explore patients’ perceptions and expectations of an upcoming orthopaedic consultation.

**Methods:**

This was an explorative qualitative study with an inductive approach. Thirteen patients who were referred for orthopaedic consultation were included using a purposeful sampling strategy. Patients participated in individual, semi-structured interviews that were recorded, transcribed verbatim and analysed with qualitative content analysis.

**Results:**

The participants’ expressed perceptions and expectations of the upcoming orthopaedic surgeon consultation were classified into 5 categories: Hoping for action, Meeting an expert, A respectful meeting, Participating in the consultation, and A belief that hard facts make evidence. Across the categories, an overarching theme was formulated: Take me seriously and do something! The participants emphasised a desire to be taken seriously and for something to happen, both during the consultation itself and as a result of the orthopaedic consultation. They described a trust in the expertise of the orthopaedic surgeon and stressed the importance of the surgeon’s attitude, but still expected to participate in the consultation as well as in the decision-making process.

**Conclusions:**

The study findings illuminate aspects that are important for patients in an orthopaedic consultation. The descriptions of patients’ perceptions and expectations can serve to improve patient–clinician relationships as well as to inform the development of new models of care, and a greater understanding of these aspects may improve the patient experience.

## Background

Musculoskeletal disorders (MSDs) are one of the main reasons why individuals consult primary health care [1]. MSDs are managed by healthcare professionals from primary care as well as specialist care; however, there are difficulties in selecting the patients that are appropriate for interventions such as surgery, as well as selecting the appropriate specialist [2]. The number of people suffering from MSDs throughout the world is expected to rise considerably over the coming decades, further increasing the burden from MSDs on orthopaedic services and healthcare systems [3]. Consequently, there is an increasing need to develop new models of care for patients referred for orthopaedic consultation; models which must be safe, accessible and efficient, and use the most appropriate healthcare professional without compromising the quality of care [4]. Physiotherapist-led orthopaedic triage is one such model of care where findings have shown consistent benefits in timely access to care from appropriately qualified healthcare professionals, who can direct patients towards the optimal treatment pathway [5]. A recent Swedish randomised controlled trial comparing physiotherapist-led orthopaedic triage with standard practice (i.e. referral straight from the general practitioner to the orthopaedic surgeon), showed significantly better selection accuracy for orthopaedic intervention and to shorter waiting times [6], good perceived quality of care [7] and no difference regarding long term follow up of patient-reported outcomes [8].

Patient experience is increasingly recognised as one of the pillars of quality in health care [9–11], and has been found to be positively associated with patient safety and clinical effectiveness in a range of MSDs, settings, and health outcomes [12].

Expectations involve the patient’s beliefs regarding the potential benefit of the treatment [13] and are likely to vary according to knowledge and prior experience [14]. Expectations have also been associated to patients’ assessment of outcome of surgery in general [15], as well as postoperative satisfaction in orthopaedic surgery [16–21].

Previous studies have described patients’ experiences of living with different MSDs [22, 23], as well as perceptions, expectations, and satisfaction with different interventions and services for a range of MSDs [24–29]. To our knowledge, no study has explored patients’ perceptions and expectations of an orthopaedic consultation. There is an increasing interest amongst both surgeons and researchers in better defining and improving understanding of patients’ expectations of orthopaedic procedures [30]. However, considering that 40% or less of patients who are referred for orthopaedic consultation are considered appropriate for surgery [31–34], it is of interest to also explore perceptions and expectations of the consultation itself. Exploring patients’ perceptions is essential to any new role that involves a shift in traditional practice boundaries [35], such as physiotherapist-led orthopaedic triage. Therefore, the aim of this study was to explore and describe patients’ perceptions and expectations of an upcoming orthopaedic consultation.

## Methods

The design used for this study was an explorative qualitative research design with an inductive approach. To strengthen rigor and comprehensiveness the study was conducted and reported according to the consolidated criteria for reporting qualitative research (COREQ) checklist for qualitative research [36].

### Setting and participants

Patients referred for orthopaedic consultation (*n* = 13) were recruited from two health care centres in the region of Västra Götaland, Sweden, during February to August 2016. A purposeful sampling strategy was used [37], with the aim of obtaining a variation of gender, age and pain location for referral. Data collection was intended to continue until no new information seemed to be forthcoming in the interviews. Inclusion criteria were: patients of working age (18–67 years) with sub-acute (4 weeks–3 months) or persistent (>3 months) pain due to MSDs, who were referred for orthopaedic consultation, with the ability to understand and speak Swedish. The exclusion criteria were based on a previously published protocol for a randomised controlled trial (RCT) [6], and were chosen in collaboration with an orthopaedic surgeon. Patients were excluded if the stated diagnosis on the referral was hallux valgus, ganglion or trigger finger.

### Data collection

The semi-structured interviews were conducted by the first author (KSS) who had postgraduate training in qualitative research methods, previous experience of qualitative research and clinical experience from treating patients with MSDs. The participants chose the location for the interviews; nine were held at a healthcare center, and four were conducted via telephone. The interviews lasted between 19 and 41 min (average 27 min), were audio-recorded and transcribed verbatim.

An interview guide was developed and agreed by all authors, to ensure that topics of interest were covered (Table 1).

**Table 1:** The interview guide

Domains
Previous experiences of orthopaedic consultation (if any): thoughts on participation, outcomes
Expectations for the upcoming consultation: thoughts on possible interventions, outcomes, fears
Perception of their own role: thoughts about participation, decision making
Perception of the role of the orthopaedic surgeon: thoughts on decision making, knowledge.
Prompts
Can you describe/explain further?
When you said __ how did you mean?
What did you think about that?

### Data analysis

Qualitative content analysis with an inductive approach was used for analysis of the data, according to the procedure described by Graneheim and Lundman [38]. The interviews were thoroughly read several times to obtain a sense of the whole. A systematic data analysis directed by the study aims was then performed, where meaning units were extracted, condensed and coded, while preserving the core. In the analysis, the codes were sorted and sub-categories and categories were developed, as internally homogeneous and externally heterogeneous as possible. This part of the analysis was still close to the data and on a descriptive level (manifest content). In the last step of the analysis the underlying meaning (latent content) was interpreted, expanding across all categories, and a general theme was formulated. The computer software NVivo 10 (QSR International Pty Ltd) was used for the analysis.

Three researchers performed the analysis in order to provide analyst triangulation and to increase credibility [38]. The first author (KSS) was responsible for coding and categorising all interviews, as well as the preliminary formulation of a theme. To verify the coding, three of the interviews were also independently coded by the other two authors (SB and MEHL), and discussed to reach consensus on coding strategy. KSS and SB discussed the codes, sub-categories and categories until consensus was reached. MEHL verified content conformity of the categories. Organisation and labelling of the categories were continually checked and modified throughout the analytical process. Lastly, the theme was constructed.

## Results

Out of 23 patients who were asked to participate, 13 patients (10 women, 3 men) accepted participation. Four patients declined to participate and six were unreachable. Participant characteristics are presented in Table 2.

**Table 2:** Participant characteristics (sorted by age)

Gender	Age	Profession	Education	Pain location on referral
Man	33	Blue collar	High school	Knee
Woman	35	Assistant nurse	High school	Thoracic back
Woman	47	Baker	High school	Shoulder
Woman	49	Office worker	High school	Neck
Woman	50	Shop assistant	2-year high school	Arm/hand
Woman	50	Postman	2-year High school	Neck
Woman	51	IT, white collar	High school	Knee
Woman	52	Orderly	Elementary	Knee
Woman	55	Assistant nurse	High school	Knee
Woman	57	Nurse	University	Foot
Man	59	Truck driver	Elementary	Hip
Man	62	Blue collar	High school	Knee
Woman	63	Teacher	University	Knee

**Fig. 1:**
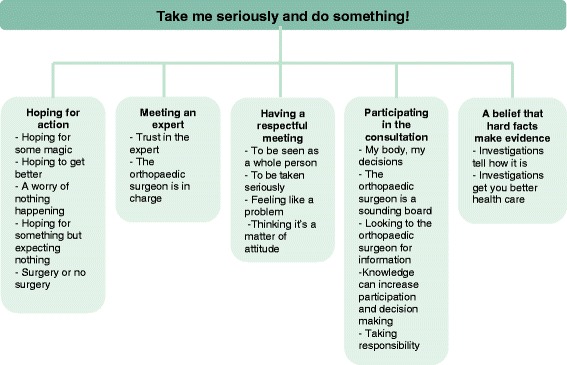
Overview of results from the qualitative content analysis with the over-arching theme, categories and sub-categories

The participants expressed perceptions and expectations of the upcoming orthopaedic consultation were classified into five categories: *Hoping for action, Meeting an expert, Having a respectful meeting, Participating in the consultation*, and *A belief that hard facts make evidence*. Across the categories an overarching theme was formulated as: *“Take me seriously and do something!”.* An overview of the results is presented in Fig. 1 An overview of the analysis is presented in Table 3.

**Table 3:** Overview of analysis with examples of meaning units, condensed meaning units, codes, categories, sub-categories and the theme

Meaning units	Well, it’s for them to say let’s do this and then you are good to go! Like waving a magic wand!	Yes, he has a very important role of course, you put your life in his hands, or her hands	Firstly, listen to what is wrong	I think that one is highly participating considering that it’s my body and that it’s me who somehow decides if I want to do what they say or not	You can see the x-ray, oh, there is a fracture here, let’s fix that. Or it’s narrow here or… well, now they have nothing
Condensed meaning units	Want them to do something, wave a magic wand	He has an important role, put your life in his hands	Listen to what is wrong	It’s my body, I decide what to do	Can see what it is on x-ray and fix it
Codes	Would like the orthopaedic surgeon to do some magic	To put one’s life in the hands of the orthopaedic surgeon	To be listened to	I am participating, it’s my body	X-ray makes it easier for the orthopaedic surgeon to know what to do
Sub-categories	Hoping for some magic	Trust in the expert	To be taken seriously	My body, my decisions	Investigations get you better health care
Categories	Hoping for action	Meeting an expert	Having a respectful meeting	Participating in the consultation	A belief that hard facts make evidence
TTheme	Take me seriously and do something!				

### Take me seriously and do something!

The theme is based on the participants’ expressed wish to be taken seriously throughout the consultation and that the orthopaedic surgeon could do that through listening, respecting, as well as by providing information and enabling the patient to participate. Moreover, an expectation of something to happen, both during the consultation and as an outcome of it, was evident across the interviews.

### Hoping for action

In this category, a feeling of wanting and hoping for something to happen during, or after the consultation with the orthopaedic surgeon was described, sometimes expressed as hoping for some magic. The participants emphasised the desire for the orthopaedic surgeon to able to do something, either wishing for surgery, or wanting to avoid surgery, but still wanting something to change.

#### Hoping for some magic

A wish for the orthopaedic surgeon to provide a quick fix for their problem was expressed. “*I believe in the magic wand you know, that they can fix this. It’s what all patients think. You want to get well, right?”* (Interviewee [I] 10). However, there was an underlying realistic sense of the fact that it does not work that way.

#### Hoping to get better

Various expectations for a positive outcome were expressed: to get rid of the pain, hoping for a recovery, to regain function.

#### A worry of nothing happening

The desire for the orthopaedic surgeon to “do something” was also manifest in a worry that the orthopaedic surgeon would not be able to do anything about the problem. *“So that would be the worst, for them to look and say that ‘you are too old, you have to live with this’.”* (I 6)

#### Hoping for something but expecting nothing

The feeling that there was a difference between what the participants hoped for, such as a diagnosis or options, and what they really expected based on previous experiences, was described. *“My hopes are that I will see someone who finally gives me a diagnosis after 18 years with broken knees. /…/ however, my expectations, after having seeked health care for soon to be 20 years, is that I will get there, and get sent home with Paracetamol and then nothing more will happen.”* (I 11)

#### Surgery or no surgery

An expectation for surgery as the best outcome of the consultation was described. *“I’m hoping for an MRI and then it was someone who said that then you just do a little arthroscopic surgery or something and then you get well again.”* (I 6)

Although participants wished for surgery, they wanted information about options, and to be part of the decision-making process. A desire to avoid surgery as long as possible was also expressed, but there was still a wish for something to happen.

### Meeting an expert

The perception of the orthopaedic surgeon as the expert was described in this category. The participants emphasised a great trust in the orthopaedic consultant, in his decisions and that he should perform his best. The orthopaedic surgeon was perceived to have a great responsibility, since the outcome of the consultation could be life changing. Also, a perception of the orthopaedic surgeon being in charge and a feeling that the patients just “had to go with it” was described.

#### Trust in the expert

All the participants expressed that they perceived the orthopaedic surgeon as the expert; that they were the ones to go to, the ones who had the knowledge and the experience. *“I’m seeing an expert. I’m not the expert, I‘m just in pain. They are the experts and the ones who should tell me what to do and to help me with that.”* (I 8). It was considered important that the orthopaedic surgeon was professional; competent, prepared and should want to solve problems. A great deal of trust was placed in the orthopaedic surgeon, both when it came to having the appropriate competence as well as decision making. *“- What would you need to accept surgery? - For them to make the decision that I need it, simple as that.”* (I 9). A feeling of the consultation being very important was described, a feeling that the outcome would have a great impact on their life. *“Yes, he has a very important role because it is still, you put your life in his or her hands.”* (I 4).

#### The orthopaedic surgeon is in charge

A perceived low level of participation during the consultation as well as in decision making was described, either expressed as not wanting to participate, not being entitled to take an active role. *“He has all the power, I think. It is him who says what you should do. I can only refuse if it sounds too bad. You have to hope that they know what they are doing, you know.”* (I 7)

### Having a respectful meeting

In this category, the importance of a respectful meeting was stressed. The participants expressed that they wanted to be seen as a whole person and to be taken seriously, and how they wished to not become a problem. The attitude of the orthopaedic surgeon was of great importance.

#### To be seen as a whole person

A wish to be seen as an individual and for the orthopaedic surgeon to take in the whole person, the bigger picture, was described. Participants expressed how they wanted the orthopaedic surgeon to be able to think new for each patient, to “reset”.

#### To be taken seriously

The importance of being taken seriously was emphasised by the participants; to be listened to and respected, to feel safe and understood. Participants hoped for a consultation where the orthopaedic surgeon did not have prejudices or ignored their problems just because they were diffuse or difficult to explain. *“That they have the perception that I am a couch potato and that I just throw myself like ‘help me’. That I am totally helpless. Not to be treated like an idiot, but to be able to have, that we are two people discussing my shoulder.”* (I 5)

#### Feeling like a problem

Some participants felt uncomfortable at the consultation and would not ask questions even though they wanted to. A feeling of taking up time for someone else was expressed.

#### Thinking that it’s a matter of attitude

The attitude of the orthopaedic surgeon was described as important. Previous experiences of a perceived bad attitude have inflicted a fear of seeking health care. Even conflicts at previous consultations were described, which was perceived to originate in the surgeon’s attitude.

### Participating in the consultation

In this category, a firm belief that the consultation was about ‘my body’ was described, and since it was ‘my body’, it was also ‘my decision’ to make regarding further actions. The orthopaedic surgeon was seen as a sounding board who was looked to for information to help with decision making. Having knowledge was expressed as a means for participation. A feeling of being responsible as a patient was emphasised, either felt as taken or assigned or forced.

#### My body, my decisions

A firm belief that the patient should be participating to a high extent during the consultation as well as in decision making was expressed, considering it is their body. *“It’s my future. My life as I have today, if I were to be one-armed for example, that would be devastating for me. Because then I can’t continue with neither my profession nor the farm. /…/ No, I am very much participating.”* (I 5).

Some patients described a possibility to refuse surgery, but also uncertainty about the possible influence if the orthopaedic surgeon should decide that surgery was not an option.

#### The orthopaedic surgeon as a sounding board

A perception of the orthopaedic surgeon role as a sounding board was expressed, where the participants themselves had an active role, and was hoping for a possibility to discuss their problem and possible interventions and outcomes. *“I just see him, or her, as a, what should I say, sounding board where they have, where they can say this is what it looks like. And then I will have to use that information and see what I want to do with it, weighing in the surgeon’s recommendation off course. Definitely.”* (I 5)

#### Looking to the orthopaedic surgeon for information

A very important role of the orthopaedic surgeon was described as being a provider of information; such as diagnosis, information about options, management and procedure. *“I mean, his approach needs to be professional and to find out what is wrong /…/ and to tell me what the possibilities are, and what we can do, how we can move on and so on.”* (I 6)

#### Knowledge can increase participation and decision making

The perception of knowledge being a factor for participation and influence was stated. *“When you have read up on stuff and done your research I think you might be able to do that. That I want, ‘I’ve heard that they have done such and such operation and I have heard that it went well, can you fix that?’”* (I 7)

#### Taking responsibility

Participants considered it important to be responsible as a patient. *“Responsibility is my own to a 100%, and yes, they are not magicians. /…/ I don’t feel, well… he’s educated of course, and gets paid to help patients, but he’s not a magician, but I hope of course, but I don’t demand him to take any responsibility for me, I am responsible for myself.”* (I 2). A general responsibility to take care of themselves was described, and a wish for advice should surgery be out of question. Furthermore, participants thought they were responsible to prepare for the consultation, to present all information about the problem, or to ask questions during the consultation. However, a feeling of having been forced to take responsibility was also described. *“I think one has to be persistent if you want to get anything done. I’m not good at that. I want them to decide what should be done.”* (I 7).

### A belief that hard facts make evidence

This category describes how investigations such as x-rays or magnetic resonance imaging (MRI) were perceived to provide evidence for the experienced problem and, if anything could be seen on these investigations, it would help you get better health care. On the other hand, a lack of these hard facts was stated as a possible barrier for being understood and helped.

#### Investigations tell how it is

Participants emphasised having a strong belief in the hard facts, i.e. results of investigations such as MRI or x-rays which were perceived as evidence. A desire to get more investigations was described, to be provided with evidence for the diagnosis. “*/.../ the best thing is if they have evidence. And now, with my knee, they want to do an MRI to conclude a diagnosis, but I can’t do an MRI because I’ve got a magnetic implant, so they won’t do it on me, and then they say, ‘but now we don’t know what it is’.”* (I 8)

#### Investigations get you better health care

The participants described a feeling that positive findings on x-rays or investigations, would get them better health care. Thus, a lack of findings would make your problem more diffuse and harder to treat. Having “hard facts”, or lacking them, was therefore perceived to influence the outcome of the consultation.

## Discussion

### Discussion of main findings

This study found that patients with MSD referred for orthopaedic consultation expressed a strong desire to be taken seriously during the consultation, and for something to happen during, and as a result of the consultation. Participants perceived the orthopaedic surgeon as an expert and were willing to place their trust in him and his decisions. However, a desire to be provided with information and options, and to participate in the consultation and in decision making was expressed. Participants also described the importance of having a good, respectful meeting with the orthopaedic surgeon. The findings are in concordance with identified core components of person-centred care, such as patient participation and involvement, and relationship between the patient and the healthcare professional [39, 40]. While all healthcare professionals provide care based on these elements, the degree to which this is done depends on the interest and priority given to these elements by the professional group [40]. The context of care for this study was the orthopaedic consultation, and recent literature has suggested that in the management of ‘preference-sensitive conditions’ such as many musculoskeletal disorders, where there is no single treatment option which clearly stands out, shared medical decision making would be especially relevant [41]. The main categories of the results in this study show similarities with previous research on perceptions and expectations for consultations or management in other settings for various MSDs [25, 26, 42–45], further elaborated on below.

The finding in this study that patients wanted something to happen, has been previously reported in a study on patients’ expectations of general practitioners management of back pain, where patients wished for more than just education and reassurance [26]. Verbeek et al. [25] showed in their systematic review of patients expectations of treatment for low back pain, that the patients expect more diagnostic tests as well as other therapy or referrals to specialists.

The perception described in this study of the orthopaedic surgeon as the expert, based on the surgeon’s experience and knowledge, is consistent with earlier studies. The expectation of proper qualifications, i.e. knowledge and skills, of the clinician has been reported both in patients seeking health care in general [44] and in patients seeing an extended scope physiotherapist [43]. The willingness of participants to place their trust in the orthopaedic surgeon, based on the view of them being the expert, as expressed in this study, is in line with the findings of Bernhardsson et al. [46] who reported a similar trust placed by patients with MSDs in the physiotherapist’s professional competence in choosing and guiding treatment. As expressed by the participants in our study, a lack of knowledge made them trust the orthopaedic surgeon with the decision making; findings that are in line with patients’ perceptions of an extended scope physiotherapist screening service [45]. This trust places a great responsibility on the orthopaedic surgeon, since the patients described a feeling of putting their life in the surgeons’ hands. It has been suggested that if patients are provided with the best available evidence regarding their disorder, as well as options for treatment, they are more likely to actively participate in their care [47], and patients in this study stated that knowledge could improve participation.

Patients in this study emphasised a desire to participate, both in the consultation as well as in decision making, and that to be able to participate they looked to the orthopaedic surgeon for information, viewing him as a sounding board. This is in line with the literature where shared decision making has been defined as an approach for clinician–patient collaboration; sharing the best available evidence to achieve informed patient preferences and reaching agreement on appropriate treatment [39, 48, 49]. Chewning et al. [50] have presented the desire for participating in decision making as a continuum, where different types can be defined; the ‘autonomist’ who wants to make decision themselves, the ‘collaborist’ who want to share decisions equally, and the ‘delegator’ who prefers leaving decisions with their healthcare practitioner. Previous studies have reported that the majority of patients want to discuss options and receive information from physicians, even though they may not wish to make the final decision [51, 52]. Patients in this study said that they wanted to participate in decision making, that it was their body, and that they wanted to participate to a high extent. This attitude seems to be in the ‘autonomist’ cluster; however, some participants assumed more of a ‘collaborator’ role, stating a preference for ‘informed consent’, and some seemed to be ‘delegators’, preferring to leave (by choice or a perception of not being able to participate) all the decision making to the orthopaedic surgeon. The participants in this study described that they were looking to the orthopaedic surgeon for information about options, management and procedure, which is supported by previous studies [43, 53]. This is in line with existing research, where shared decision-making has been defined as an approach where clinicians and patients share the best available evidence when making decisions, and where patients are supported to consider options, in order to achieve informed preferences [49]. It involves not only collaborating in making decisions about treatment, but also sharing information, building consensus about the preferred treatment, and reaching agreement on appropriate treatment [48].

The participants in our study stated taking a responsibility for their disorder; by preparing for consultation and taking an active role during the consultation. These findings are corroborated by Larsson et al. [54], who found that many patients with musculoskeletal conditions are prepared to take responsibility, both in seeking help and adhering to treatment, but that some are more inclined to share the responsibility and collaborate with the clinician.

The participants in this study emphasised that they wanted a respectful meeting and to be taken seriously. This finding is consistent with previous research on how communication is important, and how empathy and listening are closely associated with patient satisfaction and autonomy [55]. Similar to our study, it has been previously reported how patients want to be trusted and believed [42] or “being heard” [56]. Additionally, findings from recent research suggest that patients perceive clinician empathy as important [45], and that the perception of surgeon empathy during a consultation with a hand surgeon was primary linked to patient satisfaction; more so than to visit duration or pre-visit expectations of visit duration [57].

The participants in this study considered ‘hard facts’, i.e. results from x-rays or MRI, as evidence, and that the findings on these would influence the management of their problem. This is in concordance with previous research showing that patients believed that x-rays were necessary to identify the “cause of the pain”, and that any (even incidental) findings on x-ray were thought to indicate the cause of the pain [26]. It has previously been suggested that patients overestimate the benefit of tests and investigations and that by giving extensive information, patients can develop realistic expectations and make informed decisions [58].

In the tradition of qualitative research, trustworthiness of findings should be discussed in terms of credibility, dependability and transferability [38]. Several types of triangulation were used in the analytical process. A continuous dialogue amongst the co-authors was strived for throughout the data collection and in the analytic process. Although a purposeful sample strategy was used, the included participants were quite homogeneous which might affect credibility. The relatively small sample might be a limitation of the study. However, after the first twelve interviews the number of new codes emerging was low and no new information seemed to be forthcoming, and the amount of data collected was therefore judged as sufficient to answer the research question in a credible way [37]. Since the results of qualitative research are context-dependent [38], transferability of the study findings might be affected. Nevertheless, the findings in this study might be transferable to similar settings in primary as well as in secondary care/hospital settings in Sweden and internationally.

The findings illuminate aspects that are important for patients in an orthopaedic consultation, which could enhance collaboration in consultation as well as in decision making. The descriptions of patients’ perceptions and expectations can serve to improve patient–clinician relationships and a further understanding of these aspects may improve the patient experience. Since alternative models of care, such as physiotherapist-led orthopaedic triage assessment, may be considered for implementation, the results from this study could serve to inform the development of such a model of care.

## Conclusions

The main findings in this study were that patients expect to be taken seriously and for something to happen during, or as a consequence of, an orthopaedic consultation, while at the same time expecting to participate in decision making and viewing the orthopaedic surgeon as an expert and a sounding board. The descriptions of patients’ perceptions and expectations can lead to a greater understanding of these aspects, which may improve the patient experience.

## Abbreviations

I: Interviewee
MRI: Magnetic resonance imaging
MSDs: Musculoskeletal disorders
RCT: Randomised controlled trial
